# Dr. Thomas Bridgwater

**Published:** 1909-09-18

**Authors:** 


### Obituary.

The death is announced of Dr. Thomas Bridgwater, M.B., LL.D., M.R.C.S., a former medical officer at Harrow School, at the age of 85. Dr. Bridgwater qualified M.R.C.S. and L.S.A. in 1847, and five years later obtained the M.B. of London University. Forty-five years ago he was appointed medical officer at Harrow, and he held this post until his retirement in 1899. In 1888 Dr. Bridgwater received the LL.D. degree of the University of Glasgow. He had been house physician and house surgeon at King's College Hospital, and was an Associate of King's College and a Fellow of the London Medical Society. After his retirement from practice Dr. Bridgwater lived at Uckfield, in Sussex. He was a Justice of the Peace for Middlesex and Westminster.

